# The Use of Middle Latency Auditory Evoked Potentials (MLAEP) as Methodology for Evaluating Sedation Level in Propofol-Drug Induced Sleep Endoscopy (DISE) Procedure

**DOI:** 10.3390/ijerph18042070

**Published:** 2021-02-20

**Authors:** Michele Arigliani, Domenico M. Toraldo, Enrico Ciavolino, Caterina Lattante, Luana Conte, Serena Arima, Caterina Arigliani, Antonio Palumbo, Michele De Benedetto

**Affiliations:** 1 ENT Unit, “V.Fazzi” Hospital, 73100 ASL Lecce, Italy; antopal56@virgilio.it (A.P.); micheledebenedetto@hotmail.it (M.D.B.); 2 Department of Rehabilitation, Cardiorespiratory Rehabilitation Unit, “V.Fazzi” Hospital, 73100 ASL Lecce, Italy; toraldodomenico@gmail.com; 3 Department of History, Society and Human Studies, University of Salento, 73100 Lecce, Italy; enrico.ciavolino@unisalento.it (E.C.); serena.arima@unisalento.it (S.A.); 4 Wyższa Szkoła Bankowa w Gdańsku, aleja Grunwaldzka 238A, 80-266 Gdańsk, Poland; 5 Anesthesia and Intensive Care Department, “V.Fazzi” Hospital, 73100 ASL Lecce, Italy; caterinalattante@alice.it; 6 Laboratory of Interdisciplinary Research Applied to Medicine (DReAM), University of Salento and ASL (Local Health Authority) at the “V Fazzi” Hospital, 73100 Lecce, Italy; luana.conte@unisalento.it; 7 Laboratory of Advanced Data Analysis for Medicine (ADAM), Department of Mathematics and Physics “E. De Giorgi”, University of Salento, 73100 Lecce, Italy; 8 General Medicine Department, Univerzita Pavla Jozefa Safarika, 04001 Kosiciach, Slovakia; cate.arigliani10@gmail.com

**Keywords:** middle latency auditory evoked potentials, propofol, drug-induced sleep endoscopy, MLAEP index, bispectral index, obstructive sleep apnea, positive airway pressure, auditory evoked potentials, central sleep apnea

## Abstract

To analyze the middle latency auditory evoked potential index (MLAEPi), compared to the standard bispectral index (BIS), as a method for evaluating the sedation level in drug-induced sleep endoscopy (DISE). In this controlled clinical study on a sample of 99 obstructive sleep apnea (OSA) or snoring patients, we compared the MLAEPi with the BIS after propofol infusion during the standard DISE technique in order to define the MLAEPi values within the observational window of the procedure. The DISE procedure was divided into eight steps, and we collected both MLAEPi and BIS data values from the same patient in every step. The MLAEPi showed a faster response than the BIS after propofol infusion during DISE. Therefore, the clinical use of the MLAEPi in evaluating the sedation level seems to be a good alternative to the current technological standards.

## 1. Introduction

Obstructive sleep apnea (OSA) is characterized by partial or complete repeated obstructions due to the collapse of the upper airway during sleep [1], causing intermittent hypoxia, which leads to systemic damage and increases the risk of morbidity and mortality [2]. Drug-induced sleep endoscopy (DISE) is a procedure used for OSA patients to distinguish between the different obstruction types at different upper airway levels [3,4]. DISE allows for the management of surgical or positive airway pressure (PAP) nocturnal treatment failure and solving possible clinical and instrumental data mismatches [5,6]. The DISE procedure could be a reliable test [7] able to improve OSA diagnosis and treatment [6].

The main difficulty in performing the DISE procedure is reaching a well-defined sedation level [5], since the upper airway collapsibility, as a result of propofol infusion, changes according to the blood’s drug concentration [8,9,10,11,12,13,14]. The correct sedation level is indeed difficult to reach when only the clinical criteria for evaluating the sedation level are used. For this reason, an instrumental aid would be strongly required [15,16,17].

The current standard used in the clinical setting for monitoring the sedation level is the use of a bispectral index (BIS) [18], which is based on the detection of spontaneous cortex potentials. Middle latency auditory evoked potentials (MLAEPs) are known as indexes of the central nervous system depression induced by Propofol [19,20,21], and an MLAEP index (MLAEPi) is a computed index of potentials of MLAEPs. They are detected in a specific time window of auditory evoked potentials (AEP) on the auditory pathway as a consequence of a definite sound stimulus. As well as a BIS, the use of MLAEPs [22] is a valid, reliable methodology [23,24,25] that is used in anesthesia and intensive care units [26,27,28].

For testing the level of sedation, we believe that the use of middle latency auditory evoked potentials (MLAEPs) in the DISE procedure could be a better strategy compared with the use of a BIS. However, to the best of our knowledge, an MLAEPi has never been used before in the DISE technique for this goal. After having preliminarily excluded other methodologies of evaluating the sedation level, we focused on the introduction of an MLAEPi as a useful method in the DISE procedure. The MLAEPi signal is generated by a specific, stable and known input, and since we know its physical properties, we can develop the relative evoked potentials index. An MLAEPi also seems to be easier to manage, as it is a single (acoustic) pathway evoked potential compared with all of the spontaneous cortex potentials.

The purpose of this study is to assess the advantages of an MLAEPi versus a BIS in monitoring the sedation level in the DISE procedure in order to improve the diagnosis obtained by this methodology.

## 2. Materials and Methods

In the present controlled clinical study, patients were involved in the sleep apnea surgery protocol during the period from April 2015 to December 2019. We recruited 99 patients affected by snoring or OSA, as defined by the American Academy of Sleep Medicine (AASM) [29]. A total of 122 consecutive patients were enrolled, of which 23 were excluded due to missing data exceeding 10%. Accordingly, the sample size was *n* = 99 patients, with a higher proportion of men (89.69%) in respect to the women (10.31%). The mean age was about 49.8 (SD = 13.1), and the body mass index (BMI) was equal to 28.4 (SD = 3.76). The patients’ mean age reflected the enrolment criteria of the DISE procedure, which was surgically oriented and, for this reason, tended to prefer young people. As a consequence, the higher proportion of men with respect to women depended on OSA epidemiology, which is less frequent in women before menopause [30,31,32,33].

Some patients were not compliant with the PAP nocturnal treatment. We excluded participants with more than a 10% loss numerical value (effect-site propofol concentration, MLAEPi, BIS, Sat.O_2_). We also excluded pregnant women, patients with contraindications to propofol infusion and patients who refused a possible surgical treatment or had a limited mouth opening (interincisive distance ≤2.0 cm). None of the patients used antihistamine medication or other drugs able to act on the central nervous system for at least two weeks before DISE. The recruited patients had American Society of Anesthesiologist (ASA) risk classifications ≤3. All patients were found to be in the normal range after neurological clinical evaluation and were not affected by any major craniofacial anomalies. Moreover, since an MLAEPi sound stimulus was used, all recruited patients had normal hearing from at least one ear. Table 1 shows descriptive analysis of the recruited patients.

The MLAEPi and BIS data collected were analyzed by performing a *t*-test comparison with Tuckey correction between the response speed of the proposed methods and with a Bland-Altman plot to evaluate the measurement agreement of the two devices.

**Table 1:** Descriptive sample analysis.

Statistics	Age	BMI	ESS	AHI	ODI	LOS
Min	18	18	1	2.5	1	48
1st Qt	42	27	10	14.2	11.5	69
Median	52	28	12	28	21	79
Mean	49.808	28.424	11.758	32.853	22.848	76.303
SD	13.142	3.758	2.503	19.289	14.203	11.244
3rd Qt	42	27	10	14.2	11.5	69
Max	75	38	19	85	50	94

The age (y), body mass index (BMI), Epworth Sleepiness Scale (ESS), and overnight polygraphic values are defined according to the American Academy of Sleep Medicine (AASM) [29], including the Apnea-Hypopnea Index (AHI) (hour/sleep), oxygen desaturation index (ODI) (hour/sleep) and lowest saturation (LOS) (O_2_%).

### Ethics Approval

Research approval was obtained through the ethics committee of the Local Health Authority (ASL LE) at the Vito Fazzi Hospital (verbal no. 43, 3 March 2020), and informed written consent was obtained from all participants. All international ethical standards were respected throughout the study.

We used the following medical devices: (1) a flexible endoscope (rhinolaryngoscope, 11101 series, Karl Storz^®^ CDD, Tuttlingen, Germany); (2) a compatible camera (image 1 22201020, Karl Storz^®^ Tuttlingen, Germany); (3) an American Academy of Sleep Medicine (AASM)-compliant home sleep apnea testing device (Embletta Gold Portable Testing Device^®^, RemLogicE^®^ Software 2015, Embla System Inc., Broomfield, CO, USA) and its nasal canula and thermistors; (4) an oximeter (Nonin XPOD^®^, Plymouth, UK) with a finger probe; (5) a BIS system (Covidien Ireland Limited^®^, Dublin, Irland); and (6) a TCI (target controlled infusion) pump (Alaris PK^®^ by Carefusion PK, Basingstoke, UK), plus a middle latency auditory evoked potentials (MLAEPs) system (A-line^®^ sw version 1.5, owned by Danmeter A/S Denmark, Odense, Danmark). All parameters were shown in a parallel mode using the 5VsEs (5 Video stream Experimental system), that unifies all monitors used in DISE procedure up on a single monitor, already developed for this specific task [34]. The BIS range is from 100 (wide awake) to 0, with a recommended range from 80 to 60 to successfully perform DISE [5].

In this study, we divided DISE in eight steps (Table 2), and in each step, the guiding parameter was the effect site propofol concentration visible on the TCI monitor. An analytical overview of the values of the predicted effect site Propofol concentrations is given in Table 3. Such values in this study, given the crucial importance in the execution of the DISE procedure, were used as a cross-check function, but will be the object of further analytical work related to sedation in DISE. In every step, we analyzed the response of the MLAEPi versus the BIS. Beyond the propofol concentrations, to perform a reliable assessment by crossing the data, we extracted with the same method (same step, same instance and in the same patient) the obstructive pathophysiological events of the upper airways (endoscopic patterns) and systemic effects, such as the level of O_2_ saturation in the context of the polygraphic pattern.

This study simultaneously analyzed the BIS and MLAEPi methodologies to evaluate the sedation level. MLAEPi, to the best of our knowledge, has never been used before in DISE therefore except for anesthesiology patterns, we did not have a recommended range for sedation values to perform DISE. This alternative method consists of the detection of the MLAEPi, a numeric dimensionless value which ranges from 100 to 0, as well as the BIS (from 100 for wide awake to 0). The MLAEPi expresses the measurement of MLAEPs; that is to say, it expresses the window of auditory evoked potentials generated from sound stimuli detected in the medial geniculate body (MGB) and the primary auditory cortex area. The MLAEPi is related to the degree of central depression in response to the use of some anesthetics, and it was proposed as a reliable methodology for assessing the depth of the anesthesia [23,24,25]. We used the MLAEPi because it is an index which reflects the morphology of the MLAEP curves [19]. Due to its dynamicity, the morphology of the MLAEPi is difficult to be interpreted during DISE performance.

We used compatible electrodes after cleaning the skin with 70% isopropanol and accepting a value <5 KΩ after checking and monitoring impedance. The sound stimuli were monaural clicks presented through headphones. Using a specific regressive model, the MLAEPs were extracted from the row of EEG graphic spontaneous background activity in the window of 20–80 ms. The signal was cleaned by an artifact abolition system. The MLAEP index on the monitor of our machine was defined as the A-Line ARX Index (AAI)™. A proprietary software to calculate the AAI is described by Jensen et al. [35,36].

**Figure 1:**
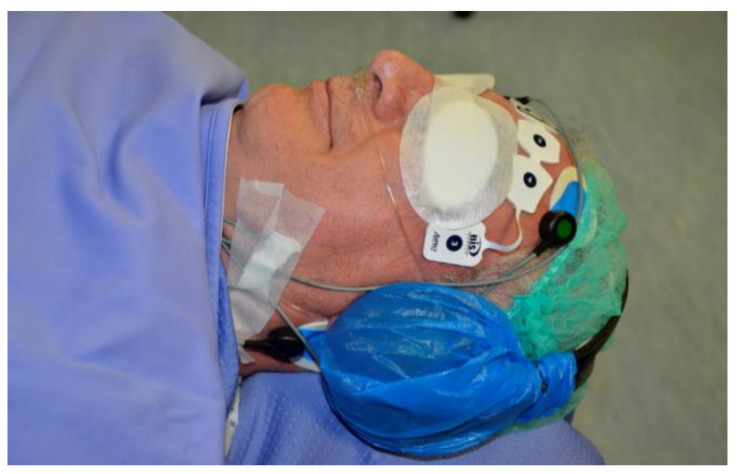
Patient equipped with headphones for the middle latency auditory evoked potential index (MLAEPi) and sensors for the bispectral index (BIS).

The MLAEPi came from MLAEP signal sampling, computed by moving the time average of the amplitude and latency. During propofol and other anesthetic administration, the MLAEP decreased in amplitude and increased in latency, so the results were a computed numerical value which decreased. The final result using the BIS and MLAEPi after the administration of propofol was a decrease in the numerical value of both, but it happened in different mechanisms and manners. We used propofol sedation administrated by a TCI pump [37,38] in order to pharmacologically reproduce the obstructive upper airway respiratory events. Figure 1 shows some of the equipment for the patient.

The BIS, MLAEPi, polygraphy and pharmacodynamic events, as well as endoscopy, were collected through our 5VsEs machine [34], which is a technological improvement of the standard DISE that permits obtaining useful multiparametric storage for post-analysis and research. All these parameters were simultaneously shown on a 5VsEs single monitor (5VsEs patent no. 202018000003044, trademark no. 302020000050401).

The procedure was carried out in a single center and in the same operation room, and it was always performed by the same operator within the same team and in accordance with the European position paper [5].

We infused Propofol in all patients that had both the most rapid onset of action and the best drug clearance properties, which are ideal for managing sedation depth during DISE. In addition, propofol provides fast post-sedation recovery. We used the predicted effect site propofol concentration measurement method through the TCI pump [39] to solve some issues dealing with the management of the typical tricompartmental pharmacodynamics of propofol. We used the Marsh algorithm [40].

For comparative data analysis, we recorded all the numerical values of the two different indexes of sedation level evaluation methods (MLAEPi and BIS), which were detected for the same patient at the same time and during equal DISE steps, with the aim of studying the performance of the MLAEPi vs. the BIS during the observation window. The timing and step definitions of data collection are listed in Table 2. Steps 3, 4, 5 and 6 represent some important moments of two obstructive respiratory events which happen once the adequate sedation level is reached. These steps, used for data collection and computing, were considered to be useful for their diagnostic value (observation window).

**Table 2:** Timing (t) and step definitions of data collection.

DISE Procedure Divided into 8 Steps:	In Every Step, We Collected the Following Data:
(1) t0-Start DISE Procedure	Predicted effect-site Propofol concentration (μg/mL) (selected from a TCI monitor) BIS value MLAEPi value O2 saturation (%) selected from a polygraphy monitor
(2) t1-Loss of Consciousness	
(3) t2-Snoring Pre-Event	
(4) t3-1st EVENT	
(5) t4-Snoring Post-Event	
(6) t5-2nd EVENT	
(7) t6-Awakening	
(8) t7-End DISE Procedure	

Steps 3, 4, 5 and 6 were considered for the observation window. As defined by the American Academy of Sleep Medicine (AASM) [29], events are patterns of upper airway obstructions characterized by oro-nasal flow characteristics, respiratory efforts and oxygen desaturation. Drug-induced sleep endoscopy (DISE), Middle latency auditory evoked potential index (MLAEPi), Bispectral index (BIS), respiratory event (EVENT).

## 3. Results

A clinical sedation evaluation was constantly performed with a supervision role [40].

**Table 3:** Predicted effect site propofol concentration values recorded in each of the procedure steps on the entire test sample.

DISE Procedure Steps	Effect Site Propofol Concentration			
	Min	Max	Mean	SD
1-Start DISE Procedure	0.00	0.00	0.00	0.00
2-Loss of Consciousness	0.40	3.20	1.70	0.54
3-Snoring Pre-Event	0.60	3.80	2.34	0.54
4-1st EVENT	1.70	4.40	2.66	0.47
5-Snoring Post-Event	1.80	4.80	2.67	0.50
6-2nd EVENT	1.40	3.90	2.48	0.55
7-Awakening	1.30	3.00	2.01	0.39
8-End DISE Procedure	1.00	2.90	1.67	0.35

Steps 3, 4, 5 and 6 were considered for the observation window. Drug-induced sleep endoscopy (DISE), Middle latency auditory evoked potential index (MLAEPi), Bispectral index (BIS), respiratory event (EVENT). Minimum (Min), Maximum (Max), Mean and Standard Deviation (SD) were reported.

Table 4 shows descriptive statistics of the two compared methods to evaluate at first glance the trends during the eight steps of the procedure. Although the two procedures were dissimilar, it was possible to highlight that the ranges of the two methods were different, particularly the range of the mean values for the MLAEPi, which was from a min = 24.81 to a max = 77.55, while the BIS method had a range with a min = 57 to a max = 95.30.

**Figure 2:**
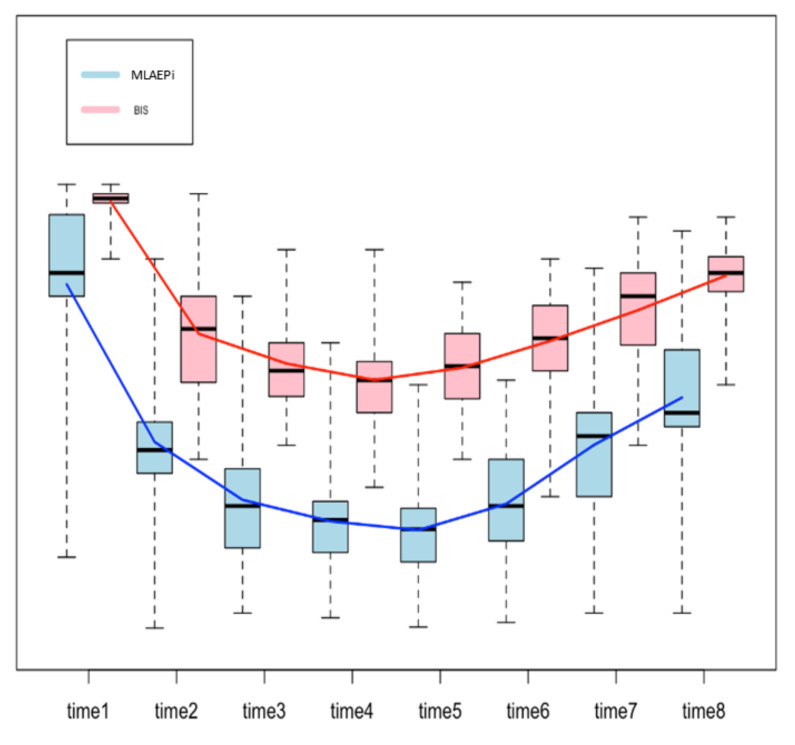
Boxplot to compare the Middle latency auditory evoked potential index (MLAEPi) and bispectral index (BIS). Since each method shows a range of the distinct means for each step, the comparison was performed on the differences between the means of the contiguous steps of analysis (e.g., procedure start mean–loss of consciousness, mean = 33.81) by using a two-sample t-test. The procedure was based on eight steps, and so there were seven differences.

Figure 2 shows the boxplots for both methods, with a line that connects the mean values from one step of the analysis to the next one. The trend of the line shows how the patients reacted to the drug, measured in terms of the MLAEPi and BIS. It is interesting to note that in the first three steps of the procedure (start of the procedure, loss of consciousness and first snoring event), the slope of the MLAEPi was greater than that of the BIS, which can be read as a fast response of the MLAEPi in the first phases. Moreover, in Steps 3, 4, 5 and 6 (which we considered the observation window), the standard deviations in the MLAEPi and BIS were quite similar, showing stability in the responses. Of course, this evaluation was only made from a descriptive point of view, and it did not take into account the two different ranges of the methods.

**Figure 3:**
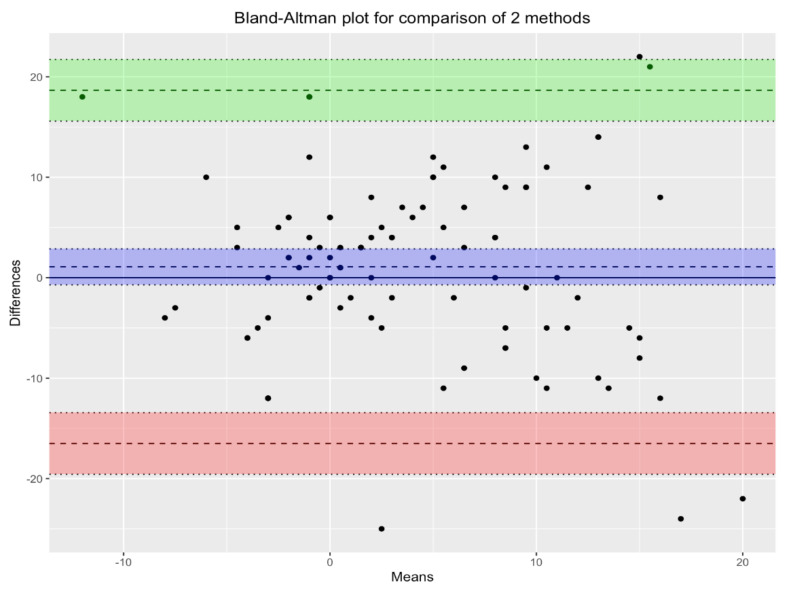
Bland-Altman plot for comparison of the two methods. There is agreement between the two devices. Violet lines define the biases of measurements. Green and pink lines define the the corresponding 95% confidence intervals.

Figure 3 shows the Bland-Altman plot for the two methods under comparison: MLAEPi and BIS. In the scatterplot, each point corresponds to a single measurement. The violet lines, which define the biases of the measurements, show the mean of the measurements with the two devices, and the green and pink lines define the corresponding 95% confidence intervals. Since almost all points lie inside the interval, we can conclude that there was substantial agreement between the two devices.

Table 5 reports the seven differences in each row, and the columns show the difference between the means of the MLAEPi and BIS in two contiguous steps, *t*-tests and *p*-values adjusted by Tuckey correction. The column of the *p*-values shows that the difference between the two methodologies was significant (*p*-value < 0.05) in the first two rows (diff t1−t2 and diff t2−t3), which applied to the first three steps of the procedures. The difference was also significant in the fourth row (diff t4−t5).

Moreover, in Steps 3–6 (the observation window), the dynamic range for the MLAEPi was equal to 54–6. In the same steps and at the same time, the BIS range was around the recommended DISE BIS interval value of 80–60.

## 4. Discussion

**Figure 4:**
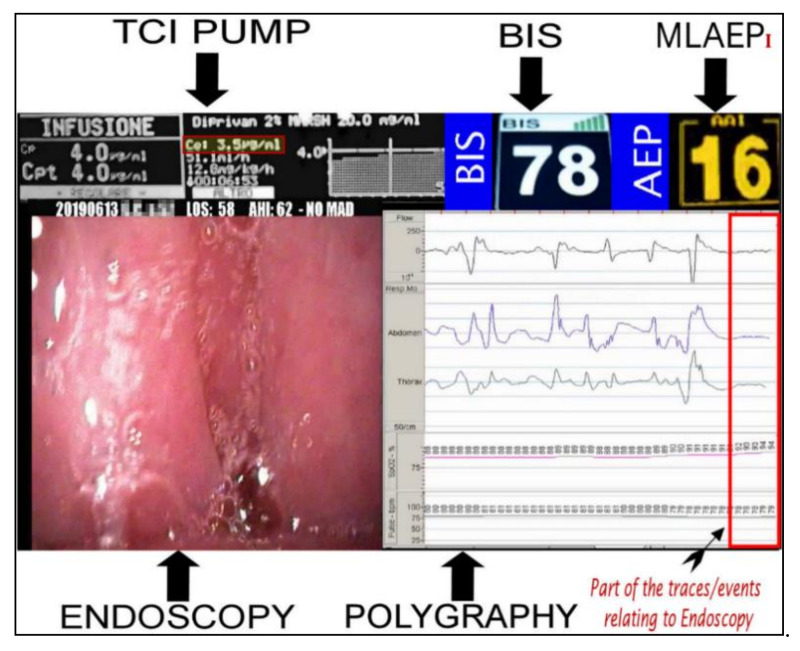
Bispectral index (BIS) and Middle latency auditory evoked potential index (MLAEPi) sedation levels from a 5VsEs (5 Video stream Experimental system) monitor. The MLAEPi value shows a real time correlation with the effect site propofol concentration (3.5 μg/mL), endoscopic pattern and the polygraphic findings of (Central Sleep Apnoea) CSA.

This study analyzed the response of the MLAEPi vs. the BIS, and the results indicated responses with different dynamics. The faster response of the MLAEPi, the most important data that we subsequently confirmed with this study, was not surprising to us, because one of the strongest starting points of this study was the theoretical advantages obtainable from an evoked potential over a spontaneous cortex potential. The visibly lower latencies of the MLAEPi response compared with the BIS led to a faster MLAEPi response occurring early in the procedure. This is of particular interest because it better shows the level of sedation in the phases approaching the observation window. This aspect seems to improve diagnosis because it avoids the diagnostic error of labeling the event shown on the monitor as obstructive apnea when it could be an undesirable propofol-induced central apnea. It is important to underline that central apneas are error sources as well as important risk factors for the ongoing DISE procedure [41]. Moreover, the MLAEPi’s faster response also seemed to lead to a better alignment to propofol pharmacodynamics. Figure 4 shows the 5VsEs [34] technology developed for this research area. The predicted effect site propofol concentration was 3.5μg /mL. The BIS value was 78. In this type of multiparametric evaluation, the MLAEPi, thanks to its speed response, was perfectly coherent with the context.

For assessing the stability and repeatability of the MLAEPi values and to disprove the suggestion that the values of the MLAEPi were too dynamic [42,43,44,45,46,47], we assessed the SD of the values from Step 3 to the end of the DISE procedure in Table 4. Similar values were found for the two indexes.

While the literature recommends a range of BIS values from 80 to 60 in DISE [5], with regard to the MLAEPi values, we still do not yet have a recommended range. The manufacturer declared MLAEPi value ranges from 100 to 0 with an intermediate value interpretation focused on the anesthesia field. For this reason, our work focused on the knowledge of MLAEPi values shown during the observation window which were already detected by BIS and validated with the clinical method [48,49]. With our instrument, we found MLAEPi values ranging from 54 to 6 in Steps 3–6 out of a total calculated sample.

Matching our results to the data from the above-mentioned literature [26,27,28] is complex, because this can be related both to the different technical specifications of the instrumentation used in previous research and to the clinical conditions different from DISE, in which an MLAEPi was used (in anesthesia and an ICU), but above all because of the peculiarities of the DISE procedure, which probably needs dedicated MLAEPi software and instrumentation born with targeted projects for DISE.

Data analysis showed dynamic MLAEPi values during the observation window which, if confirmed, might appear to be wider than the recommended range for the BIS.

Even if the MLAEPi dynamic range seems to be larger than the BIS recommended range, more studies need to be performed in order to obtain a comparative result. Overall, if these data are confirmed, they could be greatly effective, because they would make it easier for the operator to interpret the instrumental level. An MLAEPi would not only respond more quickly than a BIS but also move over a wider range.

We have noted, but at the moment cannot demonstrate, that by following only MLAEPi indications, rather than those of the BIS, the impact of human correction in the management of propofol was less frequent. These two perspectives would confirm the validity of the MLAEPi methodology.

As for the limitations of this study, although supported by 5VsEs technology, this study reported a complex data collection of about 4000 numeric values. 23 patients out of the 122 enrolled (18.85%) were excluded due to a rate of missing data higher than 10% chosen as the cut-off for the exclusion of a patient from the study. The exclusion of this cohort of patients improved the quality of the data. However, through the exclusion of these patients, we have probably lost data at particular critical moments during the procedure due to their higher difficulty in being collected. In fact, we noted that missing data were more frequently related to Steps 3 and 4 of the DISE procedure and, therefore, this indicates a critical point and a possible bias in the data collection phase.

The promising results obtained refer to the technical specifications of the machine used. The instrument used was projected for intensive care unit and anesthesiological utilization. For these reasons, although the design of this study was able to show the evolution of the sedation level in the MLAEPi vs. the BIS during the whole procedure (in each patient from t0 to t8), our data need to be confirmed in future statistical analysis. In the specific field of DISE, further studies are required to validate the DISE cut-off value and dynamic range recommended in DISE, as well as further evaluation regarding its reliability in medical practice in a larger number of patients.

## 5. Conclusions

The standards and design of this study confirmed that an index derived from MLAEPs is a useful alternative to a BIS in propofol DISE.

Through our instrument, MLAEPi values ranged from 54 to 6 during the observation window of the steps from a total calculated sample.

The most important data which emerged from this study was a faster MLAEP response compared to the BIS. This was more evident when the procedure was approaching the observation window phase, and it made the DISE procedure more reliable in diagnosis.

## Figures and Tables

**Table 4:** MLAEPi and BIS descriptive statistics.

DISE Procedure Steps	MLAEPi				BIS			
	Min	Max	Mean	SD	Min	Max	Mean	SD
1-Start DISE Procedure	19.00	99.00	77.55	20.30	83.00	99.00	95.30	3.37
2-Loss of Consciousness	3.80	83.00	43.74	15.90	40.00	97.00	67.00	12.30
3-Snoring Pre-Event	7.00	75.00	31.31	13.80	43.00	85.00	60.60	9.89
4-1st EVENT	6.00	65.00	26.72	10.90	34.00	85.00	57.00	9.39
5-Snoring Post-Event	4.00	56.00	24.81	10.90	40.00	78.00	59.70	9.71
6-2nd EVENT	5.00	57.00	30.44	12.60	32.00	83.00	65.40	10.20
7-Awakening	7.00	81.00	43.11	2.19	43.00	92.00	72.01	9.88
8-End DISE Procedure	7.00	89.00	53.26	16.18	56.00	92.00	79.42	6.40

Steps 3, 4, 5 and 6 were considered for the observation window. Drug-induced sleep endoscopy (DISE), Middle latency auditory evoked potential index (MLAEPi), Bispectral index (BIS), respiratory event (EVENT). Minimum (Min), Maximum (Max), Mean and Standard Deviation (SD) were reported.

**Table 5:** Two sample t-tests comparing the slopes of the Middle latency auditory evoked potential index (MLAEPi) and bispectral index (BIS).

Difference between Contiguous Steps	Mean of MLAEPi	Mean of BIS	*t*-Statistics	*p*-Value Adj.
diff t1–t2	33.81	28.313	2.681	0.009
diff t2–t3	12.422	6.404	5.083	0.000
diff t3–t4	4.596	3.515	1.198	0.233
diff t4–t5	1.909	−2.616	5.342	0.000
diff t5–t6	−5.636	−5.747	0.108	0.914
diff t6–t7	−12.667	−6.606	−4.431	0.999
diff t7–t8	−10.152	−7.414	−2.352	0.979

## Data Availability

The data presented in this study are available on request from the corresponding author. The data are not publicly available due to privacy and ethics.
